# Private Sector Contributions to National Malaria Surveillance Systems in Elimination Settings: Lessons Learned from Cambodia, Lao PDR, Myanmar, and Vietnam

**DOI:** 10.4269/ajtmh.22-0147

**Published:** 2022-12-19

**Authors:** Rebecca Potter, Kemi Tesfazghi, Stephen Poyer, M. James Eliades

**Affiliations:** ^1^ University of Oslo, Oslo, Norway;; ^2^ Population Services International, Vientiane, Laos;; ^3^ Independent Consultant, New York, New York

## Abstract

Comprehensive malaria case surveillance is necessary to achieve and sustain malaria elimination. In the Greater Mekong Subregion (GMS), the private sector plays a substantial role in malaria treatment. Yet, none of the six GMS countries collects complete case data from private sector points-of-care. Between 2016 and 2019, the GMS Elimination of Malaria through Surveillance program supported national malaria programs in Cambodia, Lao PDR, Myanmar, and Vietnam to execute elimination strategies by engaging the private sector in malaria case management, generating private sector case data, and integrating these data into national surveillance systems. The project enrolled 21,903 private sector outlets, covering between 52% and 80% of the private sector in targeted geographies, which were trained and equipped to perform rapid diagnostic tests (RDTs) and report malaria case data. By 2019, the private providers enrolled in the program reported a total of 3,521,586 suspected cases and 96,400 confirmed malaria cases into national surveillance systems, representing 16% of the total reported caseload by these countries (Cambodia, 25%; Lao PDR, 5%; Myanmar, 12%; Vietnam, 8%). Results demonstrated that with comprehensive support, such as training, provision of free or subsidized RDTs, first-line treatments, and routine supportive supervision, private providers can provide quality malaria case management and achieve high reporting rates.

## INTRODUCTION

The emergence of artemisinin-resistant *Plasmodium falciparum* in the Greater Mekong Subregion (GMS) posed a major threat to reducing the global burden of malaria and catalyzed countries in the GMS to develop elimination plans.^1^ By 2016, national strategic plans for malaria elimination were developed and adopted by Ministries of Health (MOH) in Cambodia, Lao PDR, Myanmar, Thailand, China, and Vietnam, with targets to eliminate *P. falciparum* malaria by 2025.

The private sector plays a significant role in malaria case management in the GMS and is the first point-of-care for up to 70% of people seeking fever treatment.^2^ The private sector for malaria service delivery is diverse, including formal and nonformal providers that provide health care services but are not owned by the government such as clinics, pharmacies, shops, and private hospitals.^3^ Regulation among private providers is variable across types, and most GMS countries lack complete, up-to-date registries on private providers. Despite global recommendations that surveillance systems should be capable of detecting and reporting malaria cases across public, private, and community-based service delivery channels, none of the six GMS countries collected complete case data from private sector points-of-care in the national surveillance system.^3^^–^^7^

Enhancements to the surveillance information system in elimination settings typically include progressing from periodic reporting of aggregated case data to real-time notification, case-based reporting, and more granular geographic data, to classify cases, inform timely response, and break chains of transmission.^3^^,^^8^ The success of strong surveillance systems for elimination of diseases is well documented in the literature surrounding smallpox eradication and elimination of polio and dracunculiasis.^9^^–^^11^ Although a breadth of literature outlines the components of robust malaria surveillance systems, few publications have described effective approaches for operationalizing these systems or for integrating private sector data.^4^^,^^7^

Between 2016 and 2019, the Bill and Melinda Gates Foundation-funded GMS Elimination of Malaria through Surveillance program, implemented by Population Services International (PSI), supported National Malaria Program (NMP) in Cambodia, Lao PDR, Myanmar, and Vietnam to execute components of national elimination strategies focused on private sector malaria case management and surveillance. The program sought to improve case detection and reporting from the private sector and integrate these data into national surveillance systems to increase the availability and use of complete case data across sectors.

This article presents case studies in Cambodia, Lao PDR, Myanmar, and Vietnam to 1) describe approaches to integrating the private sector into national surveillance systems, and 2) present and discuss results of private sector surveillance activities in those countries.

## MATERIALS AND METHODS

### Setting and population.

Between 2016 and 2019, PSI mapped, enrolled, and supported 21,903 private sector outlets in a malaria case management and surveillance program across targeted geographies in Cambodia (*N* = 915), Lao PDR (*N* = 525), Myanmar (*N* = 23,311), and Vietnam (*N* = 811). Recruitment of providers was based on epidemiology (targeting districts/townships with the highest risk according to national stratification criteria; annual parasite incidence thresholds varied by country) and outlet survey data describing the composition of the private sector for malaria case management (targeting provider types that were already engaged in malaria case management based on availability of rapid diagnostic tests [RDTs] and antimalarials).^12^ Only private providers authorized by national guidelines to provide malaria case management were recruited into the program.

“Formal private providers” included professionally trained health care personnel, including private clinics, pharmacies, general practitioners, and health cabinets. “Nonformal providers” included general retailers, sundry shops, itinerant drug vendors, and fast-moving consumer goods outlets. “Worksites” included privately owned plantations that were considered to be high risk due to proximity to forests, lack of access to nearby public health facilities and location in districts with highest annual parasite incidence rate according to national stratification.

The size and composition of private providers actively enrolled in the program fluctuated from year to year across the four country settings as some networks were scaled down as caseloads dropped, national directives for case management changed, and complementary funding sources shifted. Table 1 describes the types of private providers recruited in each country.

**Table 1:** Type of private sector health care providers recruited by country

Country	Formal private providers	Nonformal providers	Worksite-based malaria workers
Cambodia	X		X
Lao PDR	X		
Myanmar	X	X	X
Vietnam	X	X	X

### Materials.

Malaria RDTs and quality-assured first-line treatment were supplied to providers at subsidized rates or free of charge, either through national supply chains at the discretion of NMP logistics focal points or through procurement by PSI. Providers were trained to screen and test all suspected malaria cases with RDT and treat or refer confirmed cases according to national guidelines. Services were provided to patients free of charge or at a subsidized fee determined with the NMP.

### Data collection, validation, and analysis.

Private providers were trained by program staff to record malaria case data and stock data on NMP-approved standard paper registers. Case-based data disaggregated by type of diagnostic and type of parasite (*P. falciparum*, *P. vivax*, or mixed *Pf/Pv* infection) were collected for confirmed cases and included information about occupational risk factors, village of residence, and likely place of transmission. Data were transmitted via paper forms collected during monthly supervision visits or submitted electronically through mobile apps. Program staff input line-listed data for confirmed malaria cases and aggregate data on suspected cases tested into District Health Information Software (DHIS2), a free, open-source software for routine data management. Routinely reported malaria case surveillance data were reviewed and validated for each monthly reporting period by program monitoring and evaluation staff. Annual data quality assessments were conducted using a standardized tool adapted from the MEASURE Evaluation Data Quality Assessment Tool^13^ to monitor availability, completeness, accuracy, timeliness, integrity, confidentiality, and precision of routine data collection and revealed that accuracy of malaria case data reported from private sector was greater than 95%.

Data flows in each country were mapped to describe how case data were integrated into the national surveillance system in each country, including points of data validation, use of electronic reporting tools, and generation of information outputs. Program reports, surveillance bulletins, publications, minutes from meetings with NMP counterparts and public presentations of surveillance data were synthesized to provide context to the results presented.

Data were extracted from DHIS2 to present results on key surveillance indicators. Common indicator definitions were developed and configured to generate standardized analytic outputs across the four countries. Confirmed cases included only cases that received a parasitological blood test by RDTs or microscopy.

Private sector contributions to national case reporting in each country were evaluated by triangulating the number of tests and confirmed cases reported by NMPs to the WHO annual World Malaria Report (WMR) with private sector malaria case data reported through the program.

## RESULTS

Between 2016 and 2019, the private providers enrolled in the program reported a total of 3,521,586 suspected cases tested and 96,400 confirmed malaria cases into national surveillance systems. Cases detected in the private sector represented 16% of total confirmed malaria cases reported to the WMR between 2016 and 2019 by these countries.

Baseline outlet surveys conducted in 2016 revealed varying availability of diagnostic testing in the private sector (Cambodia, 75%; Lao PDR, 94%; Myanmar, 75%) and limited access to first-line malaria treatments (Cambodia, 70.9%; Lao PDR, 40.8%; Myanmar *P. falciparum* = 42.7%, *P. vivax* = 19.6%).^12^ Coverage of the private sector for malaria case management readiness (providers that had received RDTs, first-line treatments, training, supervision, and reported data) increased from 18% at baseline to 80% at the end of the program in Lao PDR in the five southern provinces; and from 3% to 62% in Myanmar. In Cambodia, baseline coverage was 9% in 2016 and the private sector was discontinued from malaria case management in 2018. Baseline data were not available in Vietnam, while end line coverage was estimated at 52% among registered private clinics within the six targeted provinces.

### Cambodia.

**Figure 1.**
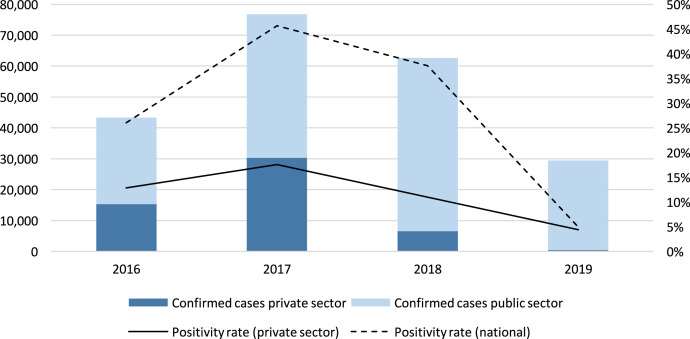
Confirmed malaria cases reported by public and private sector in Cambodia, 2016–2019.

The private sector’s contribution to the national reported caseload in Cambodia reached its highest at 39% in 2017. By 2019, the contribution of the private sector had reduced to less than 2%, following an MOH directive in 2018 that banned malaria testing and treatment services through the formal private sector and prompted the closure of the government’s Public–Private Mix (PPM) network. In 2019, 115 private worksites operating with a mobile malaria worker model (similar to the village malaria worker [VMW] program model) remained active, with substantially lowercase detection than the former PPM network. The positivity rate among private providers was substantially lower than the national positivity rate calculated according to national data reported to the WMR. Figure 1 shows public and private sector caseload trends in Cambodia. Table 2 shows private sector surveillance data in Cambodia from 2016 to 2019.

**Table 2:** Private sector testing and confirmed case data reported in Cambodia, 2016–2019

Private outlets	2016	2017	2018	2019
Private outlets enrolled in the case management program	788	856	915*	115
Confirmed cases reported by private outlets	15,263	30,219	6,575	421
Confirmed cases reported by private sector to WMR	14,847	30,214	0	0
Suspected cases that received a parasitological blood test reported by private outlets	118,521	171,719	60,063	9,527
Proportion of national caseload reported by private sector	35%	39%	11%	1%
Proportion of national confirmatory blood tests reported by private sector	71%	100%	36%	2%

WMR = World Malaria Report.

*Jan - April for PPM providers.

**Figure 2.**
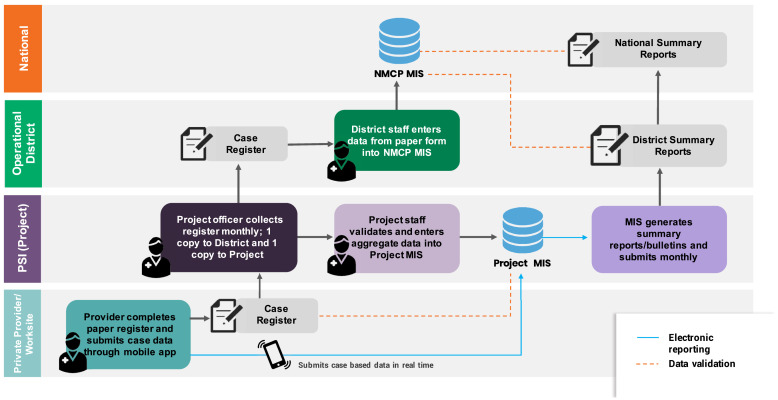
Data flow for private sector case reporting in Cambodia.

In Cambodia, annualized reporting rates ranged from 93% to 100% of expected monthly reports received in a calendar year. Worksite reporting rates presented additional challenges due to seasonal and temporary closures of worksites during certain times of the year, resulting in a fluctuating denominator of expected reports per month. Among formal private providers, the positivity rate (ranging from 12% to 19%) was consistently higher than worksite-based malaria workers (4–7%). “Zero reporting” (the submission of reports by providers that did not detect a positive case during the reporting period) was frequent. The proportion of providers enrolled in the network that detected at least one positive case in a given year ranged from 52% to 63%. Table 3 shows Cambodia’s private sector malaria case data disaggregated by private provider type.

**Table 3:** Private sector surveillance data disaggregated by formal private providers vs. worksite-based mobile malaria workers in Cambodia, 2016–2019

	2016		2017		2018		2019	
Private sector	Formal private sector (PPM)	Work-sites	Formal private sector (PPM)	Work-sites	Formal private sector (PPM)*	Work-sites	Formal private sector (PPM)	Work-sites
No. reporting units	657	131	711	129	749	121	N/A	115
Annualized reporting rate	96%	91%	99.6%	96%	92%	99%		100%
Confirmed cases reported	14,849	428	28,932	1,287	5,433	1,143		421
Cases tested	109,147	9,538	154,279	18,521	44,513	15,551		9,527
Test positivity rate	14%	4%	19%	7%	12%	7%		2%

PPM = public-private mix.

* Private health outlets were discontinued under the program during 2018 according to a Ministries of Health directive; data in 2018 reflect January–April only.

The consistency of data reported from the national malaria system to the WHO WMR compared with case data captured in PSI’s database suggests that the integration of private sector case data was highly functional. Private sector data were integrated into the national system at the operational district level. Copies of paper-based case registers were submitted to government district staff to enter into the national malaria information system at the end of each month. In parallel, data from the paper-based tools were entered into PSI’s database monthly. Electronic exports of case data from PSI’s database were extracted and shared with the NMP routinely to facilitate data triangulation and perform quality checks. Although 65% of providers were trained and equipped to report malaria case data through a mobile application, mobile reporting was done in parallel to mandatory paper reporting due to government requirements to submit paper forms to the district. The NMP and operational districts were provided with access to dashboards in PSI’s database, enabling them to access data being reported by mobile devices in real time before monthly paper reports were collected at the end of each month, collated, and entered into the national system as official data. Dashboards and surveillance bulletins generated by the national malaria system were available at district level and contained private sector data alongside data from VMWs and public facilities for use in planning activities. Figure 2 is a schematic of the data flow for private sector surveillance in Cambodia.

### Lao PDR.

In Lao PDR, case detection among the private sector more than doubled from 2017 to 2018, as the PPM network of formal private providers was reactivated and scaled up through the PSI program with subsidized diagnostic tests, case management training, and support for routine reporting. From 2018 to 2019, confirmed cases detected through the private sector fell by 55%, compared with a 44% decline in confirmed cases reported across all sectors, with steepest declines among *P. falciparum* cases in both sectors. While the national caseload declined year-over-year, the private sector’s proportional contribution to case detection increased from 5% in 2017 to 9% in 2019. Testing in the private sector steadily increased despite the declining trends in caseload, with the private sector increasing the number of tests conducted annually by more than 400% between 2017 and 2019. Tests performed in the private sector accounted for between 5% and 16% of all blood tests conducted nationally from 2017 to 2019. The test positivity rate among private providers steadily declined from 4% in 2017 to less than 1% in 2019.

**Figure 3.**
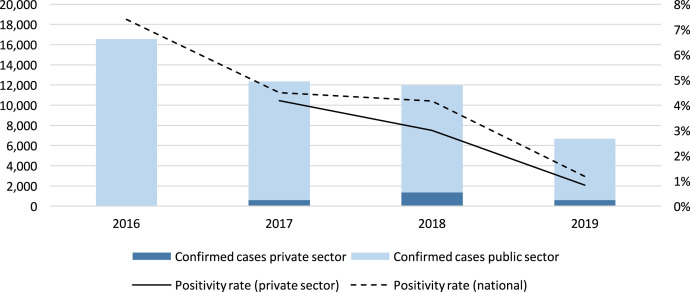
Confirmed malaria cases reported by public and private sector in Lao PDR, 2016–2019.

Annualized reporting rates among private providers were consistently high, ranging from 98% to 99%. Among providers that reported testing and case data, 39% reported at least one confirmed malaria case in 2017, and 53% detected at least one case in 2018, suggesting that roughly half of the network was reaching high-risk populations, while others detected zero cases despite consistently high testing rates. Figure 3 shows Lao public and private sector malaria case trends from 2016 to 2019. Table 4 presents private sector surveillance indicators in Lao PDR.

**Table 4:** Private sector malaria surveillance indicators in Lao PDR, 2017–2019*

Indicators	2017	2018	2019
Private sector outlets trained and equipped to report malaria case data	422	525	483
Annualized reporting rate	99%	98%	98%
Confirmed cases reported by private sector	603	1,370	612
Confirmed cases reported by private sector to the WMR	601	1,228	606
Suspected cases tested with RDT by private sector	14,424	45,611	73,754
Proportion of national caseload reported by private sector	5%	11%	9%
Proportion of national confirmatory blood tests reported by private sector	5%	16%	13%

RDT = rapid diagnostic test; WMR = World Malaria Report.

* The program launched in 2016; private networks were not activated by the program until 2017, upon the signing of a memorandum of understanding with the Government of Lao PDR. No testing or case data were reported from the private sector in 2016.

**Figure 4.**
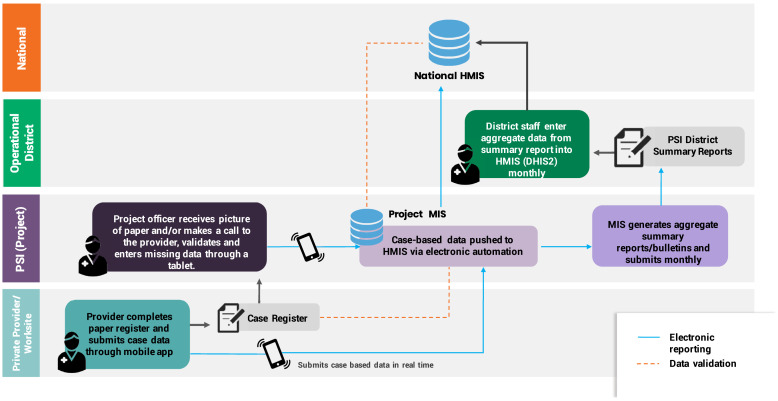
Data flow for private sector surveillance in Lao PDR.

Integration of private sector data with the national system evolved from manual district-level reporting to more sophisticated electronic data exchange of individual-level data. In 2017, PSI staff produced electronic monthly aggregated summary reports and submitted these to district health staff for manual data entry into the national health management information system (HMIS), mirroring existing reporting protocols for community health worker (CHW) data captured on paper forms. In 2018, monthly aggregate reports were replaced by electronic reporting of line-listed case data to the national system. An automated electronic data exchange mechanism was established to enable program staff to rapidly validate cases reported from private providers and push the data to the national HMIS. Robust data validation procedures by the program and feedback mechanisms between PSI program staff and NMP data focal points were essential for this interoperable solution. The NMP regularly convened malaria partners for data reviews using dashboards configured in the HMIS, with visualizations disaggregating malaria cases reported by the private sector, community, and public sector, down to district and facility levels. Figure 4 is a schematic of the data flow for private sector surveillance in Lao PDR.

Comparisons between data reported by the MOH to the WMR and PSI’s database showed that nearly 100% of the confirmed cases reported by private sector in 2017 and 2019 were accurately reported upwards from the national system, suggesting strong integration of private sector data into the national system.^14^ In 2018, there was a discrepancy of 142 cases between the WMR and PSI’s source data, for reasons unconfirmed.^15^ Interviews with program staff suggested that some case detection in 2018 through the private sector was carried out through active surveillance activities during an outbreak in the southern province of Attapeu, where pharmacists and clinics were trained to do more active screening and testing than routine passive surveillance activities. The ability to discern between routine passive case detection and active case detection by the same cohort of providers may have presented challenges to data management.

### Myanmar.

The private sector reported between 12% and 15% of annual malaria cases and accounted for between 14% and 32% of the total tests conducted in the country. Despite a 70% reduction in the size of the private sector network from 2016 to 2018 driven by the scaling down of nonformal providers, the number of confirmed cases reported by the private sector decreased by only 25% in the same period. At the same time, the private sector’s contribution to national caseload increased slightly. PSI staff in Myanmar used provider-level case data along with national stratification maps to strategically reduce the size of the nonformal network in 2018, dropping providers who had never reported positive cases.

**Figure 5.**
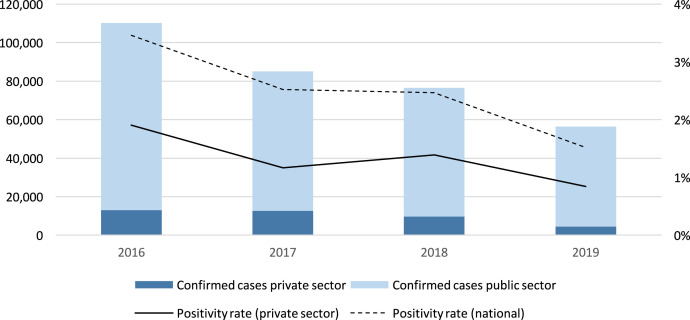
Confirmed malaria cases reported by public and private sector in Myanmar, 2016–2019.

In Myanmar, annualized reporting rates ranged from 76% to 89% of expected reports received in a calendar year among all private providers enrolled and varied across types of private providers supported. The average reporting rate was 94% among nonformal providers, 89% among community-based providers, 83% among general practitioners/clinics, and 78% among worksites. Among providers that reported case data, only 37% reported at least one confirmed malaria case, and the submission of “zero case” reports was common. The positivity rate among formal private providers was highest at 3%, while nonformal providers and worksites had annual test positivity rates between 0.5% and 1.5%. Figure 5 shows trends in case reporting and positivity rate across public and private sector. Table 5 outlines private sector surveillance indicators in Myanmar.

**Table 5:** Private sector malaria surveillance indicators in Myanmar, 2016–2019

Indicators	2016	2017	2018	2019
Private sector outlets trained and equipped to report malaria case data	20,133	15,921	6,108	6,727
Annualized reporting rate	87%	89%	76%	78%
Confirmed cases reported by private sector	13,015	12,580	9,718	4,388
Confirmed cases reported by private sector to WMR	3,030	2,599	2,216	2,257
Suspected cases tested with RDT	683,072	1,076,127	698,081	520,342
Proportion of national caseload reported by private sector	12%	15%	13%	8%
Proportion of national confirmatory blood tests reported by private sector	21%	32%	23%	14%

RDT = rapid diagnostic test; WMR = World Malaria Report.

**Figure 6.**
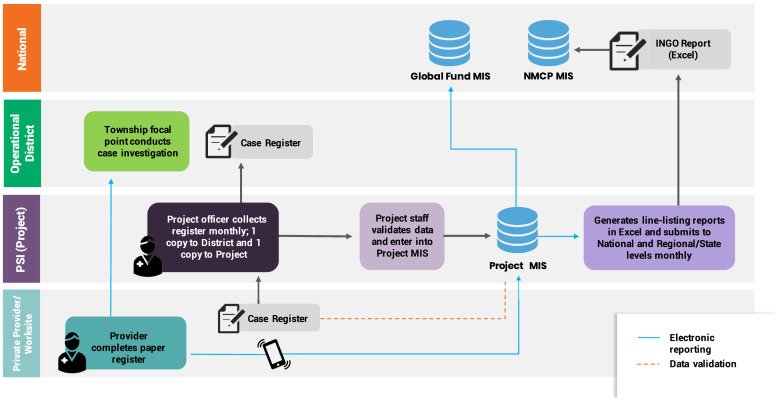
Data flow for integrating private sector data into national system in Myanmar.

Standard NMP paper registers were used and these data were manually entered into the database to generate the monthly Excel reporting sheets for NMPs. Electronic reports were generated on a monthly basis from the PSI database following data validation and quality checks, then submitted to the national, state/regional, and township levels as Excel sheets for importing into the national surveillance system following protocols for all implementing partners supporting VMW networks. In 2019, as elimination protocols were introduced in selected regions and townships with API less than 10, private providers were trained and equipped to follow case notification procedures, submitting photos of case notification forms by mobile device to the designated NMP district focal point. Figure 6 is a schematic of the data flow for private sector surveillance in Myanmar.

Private sector case data reported to the WMR reflected a small proportion (between 21% and 23%) of the total number of confirmed cases reported by private sector providers data captured in PSI’s database^14^^–^^17^ A portion of PSI’s nonformal malaria provider network in Myanmar is considered by the NMP to be reporting from a community-level place of care and unlikely to be included in the private sector reporting to the WMR, which may partially explain the discrepancies. Underreporting of private sector case data may be reflective broader challenges in Myanmar for consolidating data reporting from many implementing partners, across all sectors.

### Vietnam.

From 2017 to 2019, the proportion of cases reported by the private sector increased steadily from 3% to 17% and the raw number of cases reported increased by 77%. By comparison, the malaria cases in Vietnam dropped by 31% in the same period. Private sector case data were reported from 330 private clinics in 6 high-risk provinces, located primarily near forests, compared with 11,000 public commune health centers and 1,150 public hospitals. Meanwhile, the private sector contributed less than 1% of total diagnostic tests in the country and had test positivity rates nearly 5% points higher than the national average. This suggests the targeting of private sector providers for malaria case management in areas of highest risk was effective in the context of low burden Vietnam, notwithstanding that testing is generally high in the public sector.

**Figure 7.**
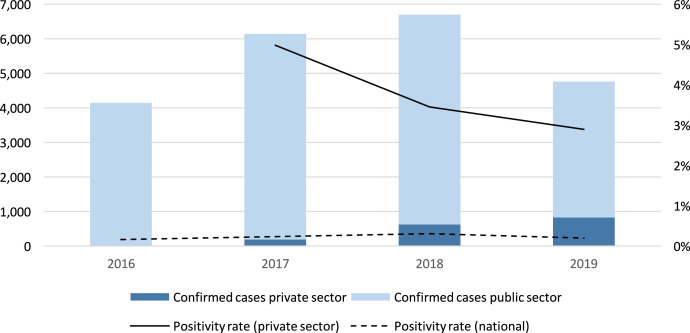
Confirmed malaria cases reported by public and private sector in Vietnam, 2016–2019.

Annualized reporting rates increased year-over-year from 75% in 2017 to 100% in 2019. The proportion of providers that reported at least one confirmed case in a calendar year increased from 18% in 2017 to 39% in 2019. Figure 7 shows trends in case reporting and positivity rate across public and private sector. Table 6 outlines private sector surveillance indicators in Vietnam.

**Table 6:** Private sector malaria surveillance indicators in Vietnam, 2017–2019

Indicators	2017	2018	2019
Private sector outlets trained and equipped to report malaria case data	164	225	330
Annualized reporting rate	75%	97%	100%
Confirmed cases reported from private sector	188	626	822
Suspected cases tested with RDT	3,771	18,152	28,422
Proportion of national caseload reported by private sector	3%	9%	17%
Proportion of national confirmatory blood tests reported by private sector	0%	1%	1%

RDT = rapid diagnostic test.

**Figure 8.**
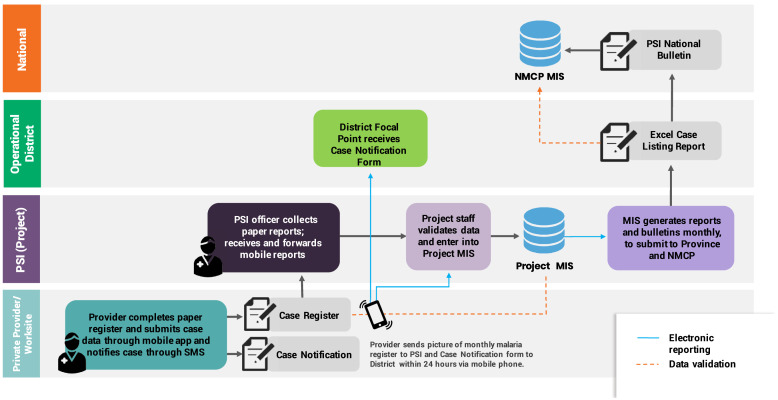
Data flow for integrating private sector data with national malaria system in Vietnam.

Case data were recorded on paper forms by private clinicians and typically sent to program staff through a photo taken by providers using their own personal devices. Mobile device ownership among this group was extremely high, and mobile credits were provided as an incentive for providers to use their devices for reporting. Line-listed case data were entered into PSI’s database and extracted monthly into an NMP standard Excel report used by all implementing partners in the country, and submitted electronically to the provincial level. Figure 8 is a schematic of the data flow for private sector surveillance in Vietnam.

Vietnam reported private sector cases to the WMR for the first time in 2018 (*N* = 39 cases), compared with the 626 cases detected by private providers and reported through PSI to provincial health authorities.^15^ In 2019, 100 malaria cases were reported in the WMR, compared with the 822 in PSI’s database.^14^ Underreporting of private sector cases may reflect a nascent malaria information system in Vietnam, where private sector reporting to the NMP had never been introduced prior to 2017.

## DISCUSSION

As countries shift from malaria control to elimination, the value of timely, case-based data from all sectors in areas of local transmission is increasingly important to ensure that every case is notified, investigated, and classified to inform timely response. Results demonstrate that the private sector can achieve high reporting rates of malaria testing and case data when incorporated into a holistic support program for malaria case management. This was also true among nonformal providers, such as shopkeepers, sundry shops, and itinerant drug vendors in Myanmar, which had higher reporting rates than their formal sector counterparts. Core interventions for the private sector are not substantially different from those used by the public sector, such as VMW models. The training, free and subsidized RDTs and first-line treatments, and routine supportive supervision received by providers is likely a contributing factor to high reporting rates. While the reliance on these program staff represented a significant resource requirement, a separate costing study that used financial data from the program in Cambodia, Lao PDR, and Myanmar found that private sector surveillance interventions were affordable when analyzed alongside costed national strategic plans for malaria elimination.^18^ These results along with other literature from the GMS suggest that networks of private providers engaged through NMP public–private partnership programs can be strengthened as a pathway to sustainable surveillance, if properly resourced.^19^

In settings where the public sector alone cannot adequately cover the population at risk and where high-risk populations face barriers to accessing care at public facilities or from VMWs, the private sector may be a crucial partner in the malaria elimination end game.^20^ Among some populations in malaria endemic areas of the GMS, fever care is sought primarily through the private sector.^2^^,^^12^ Although case detection through the private sector gradually declined in Cambodia, Lao PDR, and Myanmar, in Vietnam, the private sector’s case detection and contribution to national caseload increased year-over-year from 2017 to 2019. In Lao PDR A relatively small number of targeted private sector service delivery points (330 compared with 10,000 + public facilities that provide testing) were highly effective in detecting cases, reporting up to 17% of the national caseload. Further research synthesizing health-seeking behaviors among high-risk groups and surveillance data should inform strategies for case detection at the last mile.

Highly granular case data geolocated to the point of case detection was used effectively by program staff to adjust network composition, size, and scale. In Lao PDR and Vietnam, providers with high caseloads were identified and provided follow-up visits to understand why their outlet seemed to be reaching higher risk populations and provide differentiated program support. Funding cuts in Myanmar forced the reduction of the nonformal provider network by 62% from 2017 to 2018; yet the raw number of cases detected dropped by just 23%, even within the context of a national decline in cases. NMPs can use similar approaches to target private sector interventions and allocate resources more effectively for case-finding among high-risk groups as the malaria map shrinks. However, challenges remain integrating the provider-level granularity into national systems which typically contain geolocated master facility lists for the public health facilities only.

Although not all are unique to the private sector, barriers to integrating private sector data into national reporting systems range from gaps in accurately identifying the composition of the private sector actors, challenges in regulating the private sector, public health systems designed and implemented with public sector providers with limited input from the private sector, gaps in capacity to provide quality malaria case management, and the unique nature of the private sector set up.^4^^,^^7^^,^^21^ Enumerating and mapping private providers, as recommended by the WHO’s malaria surveillance manual, proved costly and challenging in the absence of complete, up-to-date registries of formal private providers, such as clinics and pharmacies, and far less information was available on the number and distribution of nonformal providers, such as sundry shops.^3^ The number of private sector outlets operating in the GMS fluctuated substantially due to seasonality, temporary closures, relocation, and other issues. These fluctuations make it challenging to maintain oversight of operations and calculate reporting rates, and they present substantial challenges for NMPs to properly model and maintain private sector reporting units in the national surveillance system over time. A more holistic approach to oversight of the private health sector and collaboration with other agencies such as Food & Drug Agencies and regulatory agencies to maintain comprehensive registries of private practitioners, pharmacies, and drug shops would likely be more sustainable and cost effective than tackling these issues alone for malaria programs.

Utilizing hybrid paper and electronic reporting mechanisms as part of a national scale electronic information system have proven effective in other settings, such as Thailand.^22^ Despite investments in scaling up mobile tools in the GMS for real-time reporting, the vast majority of decision making by program staff and by NMP staff in the four countries studied continues to be based on source data captured by monthly paper-based registries.^21^ While custom smartphone applications for reporting and donor-funded devices are common interventions, less attention has been paid to providers’ informal use of social media apps and routine phone calls to report case data. This phenomenon was observed across networks of private providers in the GMS for routine monthly reporting in Vietnam and Laos as well as real-time case notification in elimination townships in Myanmar. Investments in equipping the private sector for case reporting should be further considered in an evolving context of mobile coverage, smartphone ownership, and an understanding of providers’ behaviors and preferences.

The transition into a fully integrated malaria surveillance system that reflects data from the public and private sector will depend on the capacity and resourcing of the public sector health system. The Lancet Commission on Malaria Eradication emphasized the importance of improving inexpensive and readily adoptable approaches to strengthen the management, operational precision, and effectiveness of malaria elimination programs.^23^ Successful integration of private sector data into the national system in Cambodia and Lao PDR was highly dependent on 1) governance and data management Standard Operating Procedures (SOP) established at central level, and 2) linkages to district level public sector health structures. Cambodia and Lao PDR had stable national malaria information systems and private sector case data were able to be captured into those systems at the district level through existing SOPs, working with established data focal points. Implementing partners actively participated with district and national staff in data quality reviews, data analysis, and triangulation. In Vietnam, the provincial health authorities were key partners for delivering joint training and establishing reporting protocols from the private sector for the first time. Case notification procedures for the private sector were established in Myanmar with township and district focal points. The need for management capacity and demand for data to inform decision-making is crucial not only at national level, but with a focus on district health offices. At the last mile, establishing linkages between private providers and health facilities, as is common with CHW and VMW programs may facilitate more sustainable reporting flows.

Enacting mandatory case reporting policies and guidelines is necessary but not sufficient to ensure timely and quality data reporting from the private sector. There is limited evidence on the private sector’s capacity and motivation to report malaria cases data directly into government systems in the absence of a comprehensive malaria case management and surveillance support. program.

However, a recent study demonstrates that private sector providers are motivated by a complex range of internal and external factors such as commitment to serving their community, boosting their reputation, having access to professional development opportunities, and receiving commodities.^20^ These factors along with the lessons from the PSI experience in the GMS can be leveraged to refine context-specific strategies for incorporating the private sector into national surveillance systems in elimination settings.

## CONCLUSION

In the GMS, the private sector is capable of achieving high reporting rates of malaria testing and case data when incorporated into a holistic support program for malaria case management that includes training, affordable access to RDTs and first-line treatments, and supportive supervision. The private sector detected a substantial proportion of the total reported caseload in the GMS between 2016 and 2019: Cambodia (25%), Lao PDR (5%), Myanmar (12%), and Vietnam (8%). Integration of private sector data into national malaria surveillance systems is possible when interventions are designed with NMPs and aligned to existing operational procedures in place for routine public sector and community-based service data. Variances between trends in case detection and positivity rates between the public and private sector data suggest that smart targeting of private sector outlets for malaria case management may improve detection among certain high-risk populations in low transmission settings. Results demonstrate that case notification from the private sector is possible and feasible within elimination contexts.

### Limitations.

The study relied on routine data reported from health providers. There are assumptions that cases from the project are those reflected in government reporting to the WMR based on the program’s unique role of providing private sector support during the years included in the study. It is also possible that there are discrepancies between the malaria case data reported to the WMR and data captured in national surveillance information systems.
